# Magnetic Trampoline Resonators Made of (La,Sr)MnO_3_ Single-Crystal Thin Films

**DOI:** 10.1021/acssensors.5c00448

**Published:** 2025-06-06

**Authors:** Nicola Manca, Dhavalkumar Mungpara, Leonélio Cichetto, Alejandro Enrique Plaza, Gianrico Lamura, Alexander Schwarz, Daniele Marré, Luca Pellegrino

**Affiliations:** † CNR-SPIN, C.so F.M. Perrone, 24, Genova 16152, Italy; ‡ Institute of Nanostructure and Solid State Physics, University of Hamburg, Hamburg 22761, Germany; § Dipartimento di Fisica, Università degli Studi di Genova, Genova 16146, Italy

**Keywords:** magnetic sensors, mechanical resonators, mechanical sensors, oxides, MEMS

## Abstract

Microelectro-mechanical resonators employing a magnetic element have been proposed for magnetic field sensing applications, but the integration of magnetic materials with standard semiconductor compounds is challenging and requires complex fabrication protocols. We present a different approach relying on (La_0.7_,Sr_0.3_)­MnO_3_ (LSMO), an oxide compound that works both as a structural element for the resonator and a functional magnetic layer. Suspended trampolines are realized in a single-step process from LSMO thin films and show a quality factor of up to 60k and *f*·*Q* products reaching 10^10^ Hz. Their magnetic properties are probed by a SQUID magnetometer and magnetic force microscopy, showing a saturation magnetization of 240 kA/m at room temperature and in-plane magnetic domains with a coercivity of 2.5 mT. Being entirely made from a magnetic material, these resonators exhibit a larger magnetic interaction volume compared to other solutions, making them ideal candidates as building blocks for high-sensitivity magnetic field sensors.

## Graphical abstract

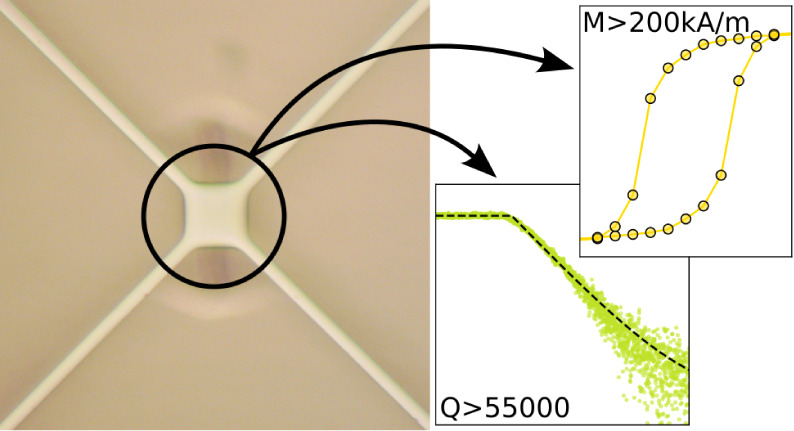

## Introduction

Micromechanical resonators are ideal transducers for applications requiring high sensitivity.^1,2^ This is due to the precision of mechanical frequency measurements and the continuous improvement in the mechanical quality factor (*Q*) of the resonating structures.^3,4^ Simple cantilever or microbridge resonators can be easily fabricated in large arrays and are described by analytical functions, making them the preferential choice for many prototypical device applications.^5–9^ On the other hand, low mechanical losses, which result in higher *Q* values, are typically achieved by combining high tensile strain and complex geometries, such as hierarchical structures, perimeter-mode resonators, or membrane-based phononic crystals, leading to *Q* > 10^9^ at room temperature.^10–13^ Suspended trampolines fall between these two categories. They consist of a central pad anchored by thin tethers to an external frame—a design that allows for high tensile strain while balancing geometric complexity and mechanical performances.^14^ Trampoline resonators have been considered in a variety of sensing experiments, including displacement detectors,^15^ THz bolometers,^16^ free-space opto-mechanics,^17^ pressure sensors,^18^ or room-temperature quantum opto-mechanics.^19^ Mechanical detection techniques have also been employed for developing high-sensitivity magnetometers,^20–23^ and most of them rely on the integration of magnetic materials with mechanical resonators based on standard semiconductors. These semiconductors are the best choice for high-*Q* mechanical resonators due to reliable fabrication protocols, excellent material quality, and integration with existing CMOS technology.^17,24–27^

However, the integration of magnetic materials with Si-based MEMS is challenging and typically requires complex fabrication protocols. A different possible approach consists of designing micromechanical devices from materials inherently showing magnetism, such as (La,Sr)­MnO_3_ (LSMO), a transition metal oxide (TMO). LSMO shows high saturation magnetization and an easy magnetization direction that can be controlled by shape anisotropy.^28^ LSMO microbridge resonators show high tensile strain and *Q*-factor in the range of a few tens of thousands,^29^ so this compound is a candidate for the realization of more complex TMO-based micromechanical magnetic sensors, particularly LSMO-based trampolines. As an example, an ultrasensitive magnetic field sensor relying on the coupling between a micromechanical magnetic sensor and a high-Tc superconductor field-to-gradient converter^30^ has been recently proposed, and a possible implementation of the aforementioned device could be realized in a full-oxide heterostructure comprising a superconducting circuit and an LSMO mechanical resonator. Moreover, a resonator fully based on a complex oxide allows for the integration of other oxides, such as piezoelectric/dielectric elements, phase-transition materials, or even high-Tc superconductors^31^ into a single crystalline heterostructure to realize multifunctional devices. Having epitaxial bilayers and heterostructures is also an important factor to achieve better mechanical coupling, high resistance to peel-off, and physical coupling between interfaces

In this work, we discuss the fabrication of magnetic trampoline resonators in view of their applications in mechanical magnetometers that work at room temperature or liquid nitrogen. Mechanical properties of LSMO trampolines, having a total diagonal of up to 700 μm, were characterized in terms of stress and quality factor, and the results were compared to numerical simulations. Magnetic properties of bare LSMO thin films are measured by SQUID magnetometry, while the magnetic microstructure of LSMO trampolines is investigated by room-temperature field-dependent magnetic force microscopy. The trampoline geometry allows us to obtain a relatively large magnetic volume (the pad) while basically preserving the advantage of a quite simple geometry in terms of design and fabrication yield. Also, in the trampoline geometry, the pad (the functional magnetic element) moves parallel to the surface and does not bend significantly, as in the case of microbridges, membranes, and cantilevers. This is an important factor for interferometric detection schemes and, in general, for optomechanical devices..

## Results and Discussion

### Device Fabrication

Epitaxial (La,Sr)­MnO_3_ thin films with a thickness of 100 nm were grown by pulsed laser deposition on 5 × 5 mm^2^ single-crystal SrTiO_3_(110) substrates, as reported in the Experimental Section. Structural analysis by X-ray diffraction is reported in the Supporting Information Section I. The main steps of the fabrication process of LSMO trampolines are illustrated in Figure 1a and follow the protocol already employed in ref 29. The trampoline geometry is transferred to the LSMO thin films by UV lithography, followed by physical etching by Ar ion milling. The trampolines are then suspended by immersing the sample into a 5% HF water solution, which corrodes the STO substrate without affecting the LSMO layer.^32^ Details of the fabrication protocol are reported in the Experimental Section. Optical micrographs of a trampoline pad during the wet etching process are reported in Figure 1b. These images were acquired every 10 min by taking the sample out of the acid bath and keeping it in deionized water, illustrating the progression of the trampoline’s release from the substrate. Clamped regions appear dark/purple, while the suspended LSMO film appears light/yellow. The tethers have a width of about 4 μm and become suspended in about 20 min, while the pad, a square of a 20 μm side, is fully released in less than an hour. STO etching anisotropy determines an asymmetry in the pillar below the squared pad during the release, which progressively becomes rectangular or even butterfly-like. Once the release is complete, this pillar leaves a ridge below the trampoline, which is oriented parallel to the [001] lattice direction (see the rightmost panel of Figure 1b). This is also visible in Figure 1c, showing an optical micrograph of an LSMO trampoline having a total diagonal of 700 μm after being removed from the acid bath and dried in a critical-point dryer system. Here, the surface of the pit between the tethers is flat and maintains its original [110] orientation, with no faceting, because of the STO etching anisotropy.^33^ The black regions at the edges of the trampoline pit are flat surfaces that do not reflect light back into the microscope objective and are due to the faceting of the STO during etching. These regions are also visible in the scanning electron microscope image reported in Figure 1d.

**Figure 1:**
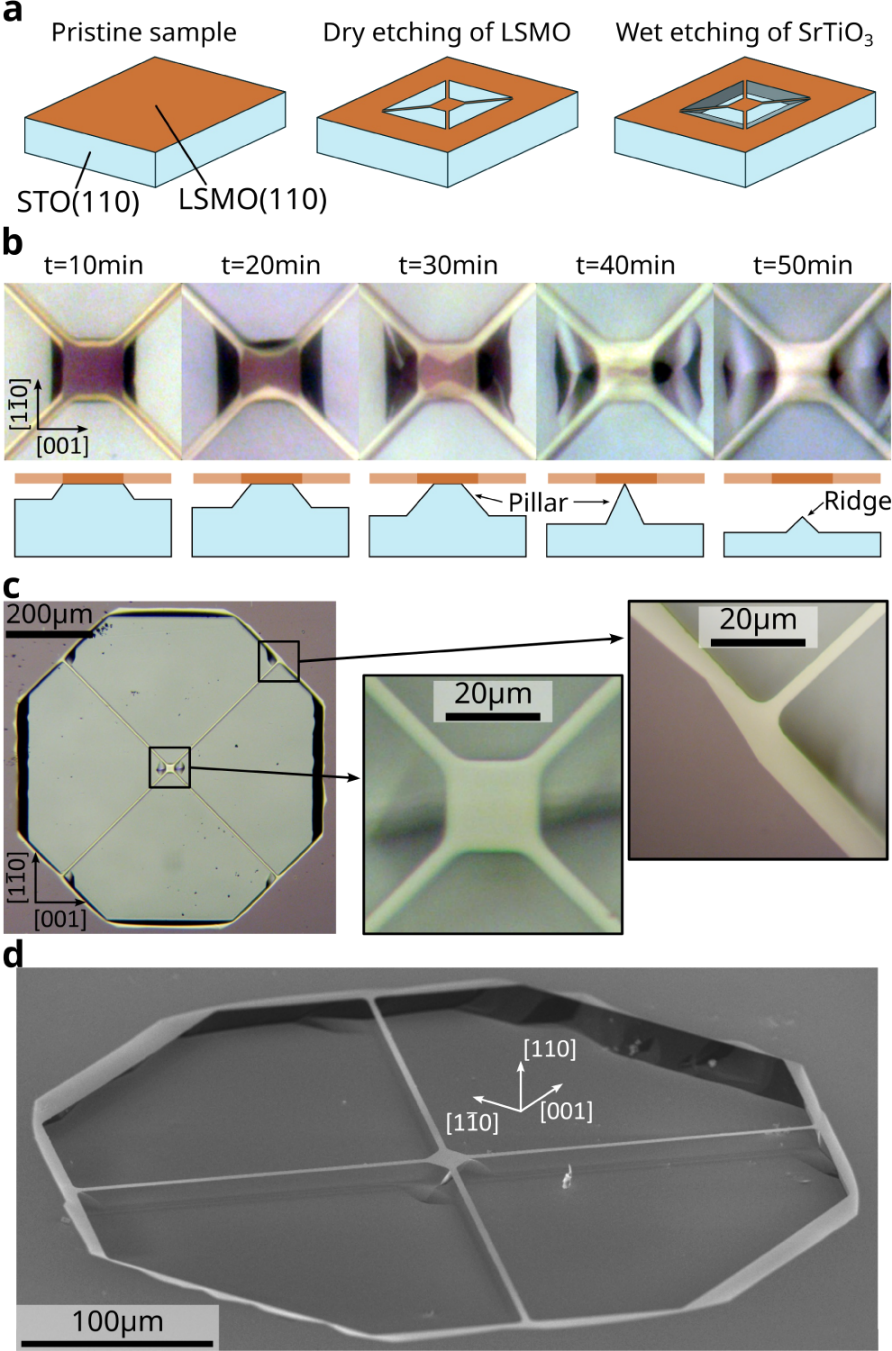
Fabrication of LSMO trampolines. (a) Main steps of the trampolines fabrication process. (b) Optical micrographs in reflected light of the pad (20 × 20 μm^2^) of a LSMO trampoline showing the time evolution of the release process. Below each picture, we included a schematic drawing of the vertical cross-section of the device. (c) Optical micrograph of a 700 μm LSMO trampoline after the fabrication process. The pad is a 20 μm square, and the tethers’ width is 4 μm. (d) Scanning electron microscope image of a LSMO trampoline.

In-plane etching anisotropy of STO(110) is crucial in determining the release process and should be considered when designing the tether geometry. This is because etching rates and walls profiles along the [001] and [11̅0] directions are different, and tethers aligned along these two axes have different suspension times and clamping conditions.^34^ By designing the tethers rotated 45° in-plane, we ensure they are all suspended at the same time and with the same clamping point geometry (except for mirror symmetry). Anisotropic under-etching is better visible when looking at the perimeter of the trampoline pit. The edges parallel to the [001] direction (the top/bottom ones in Figure 1c) show about 20 μm of under-etching, while those parallel to the [001] direction (the left/right ones in Figure 1c) show just a few micrometers of under-etching. The edges parallel to the four [11®1] equivalent directions show under-etching of about 10 μm, a value in between the two previous cases. Different clamping geometries could be obtained by designing the clamping points along edges with different alignments, for example, by choosing the direction where the under-etching is lower. However, lower symmetry in the tether configuration may result in a deformed structure upon release due to stress relaxation. The interplay between the geometry of resonators with more than two clamping points and their mechanical properties is of great interest for ultrahigh quality factor Si-based resonators^10,12^ and represents a future research direction for full-oxide mechanical devices as well.

### Mechanical Characterization

Mechanical properties of LSMO trampolines were probed by measuring the resonance frequency and quality factor of devices with different sizes. Details of the experimental setup and measurement protocol are reported in the Experimental Section. The characteristic length for the trampolines is the distance between two opposite tether clamping points, i.e., the pit diagonal. Since no analytical solution exists for the modes’ frequencies of a trampoline resonator, we performed a numerical simulation by finite element analysis to calculate the resonance frequencies as a function of the stress (σ) for different pit diagonal (*d*) values. Details of the simulation are reported in the Experimental Section. Results for the frequencies of the first mode are shown in Figure 2a, where the panel inset shows the corresponding mode shape. These data are employed to evaluate the stress of the trampolines from the frequency measurements, as discussed below. Figure 2b shows the first-mode resonance frequency measured on a set of LSMO trampolines. Here, the gray dashed lines are constant-stress curves in the frequency–length space parameter. They are spaced 100 MPa apart and correspond to vertical cuts of Figure 2a. By comparing the measured frequencies with the numerical simulations, we obtain the corresponding in-plane stress reported in Figure 2c. To do so, for each measured device, we look for the stress value in Figure 2a that provides the closest-matching frequency. These numerical solutions were calculated with a step size of 5 MPa, which allows us to evaluate the stress of the trampolines with reasonable precision. Although the limited number of measured devices does not allow for a robust statistical analysis, the average stress magnitude is about 300 MPa. These values are in good agreement with previous measurements performed on LSMO microbridges, although here few devices show stress reaching 400 MPa, which is the highest value reported so far for oxide resonators.^29,34^ The origin of such a high value could be related to slightly different local growth conditions, such as temperature gradients or alignment of the sample with respect to the plasma plume, suggesting that tensile stress may be improved by tuning the growth conditions. We measured the quality factor of the first mode of the trampolines from ring-down measurements. The experimental data of the trampoline having the highest *Q*-factor is shown in Figure 2d, corresponding to a *d* = 700 μm device. Figure 2e shows the *Q*-factor of all the resonators reported in Figure 2a. They show a steady increase in the *Q*-factor starting from 400 μm of diagonal and getting close to 60k for 700 μm ones. The linear increase of the *Q*-factor with size observed for *d* > 400 μm indicates that the mechanical losses are dominated by dissipation-dilution mechanism, suggesting that LSMO intrinsic *Q*-factor could be obtained by realizing microbridge resonators in this length range.^4^ From these data, it is possible to evaluate the *f*·*Q* product of LSMO trampolines, which is reported in Figure 2f. For all the measured trampolines, these are just below 10^10^ Hz and show no size dependence.

**Figure 2:**
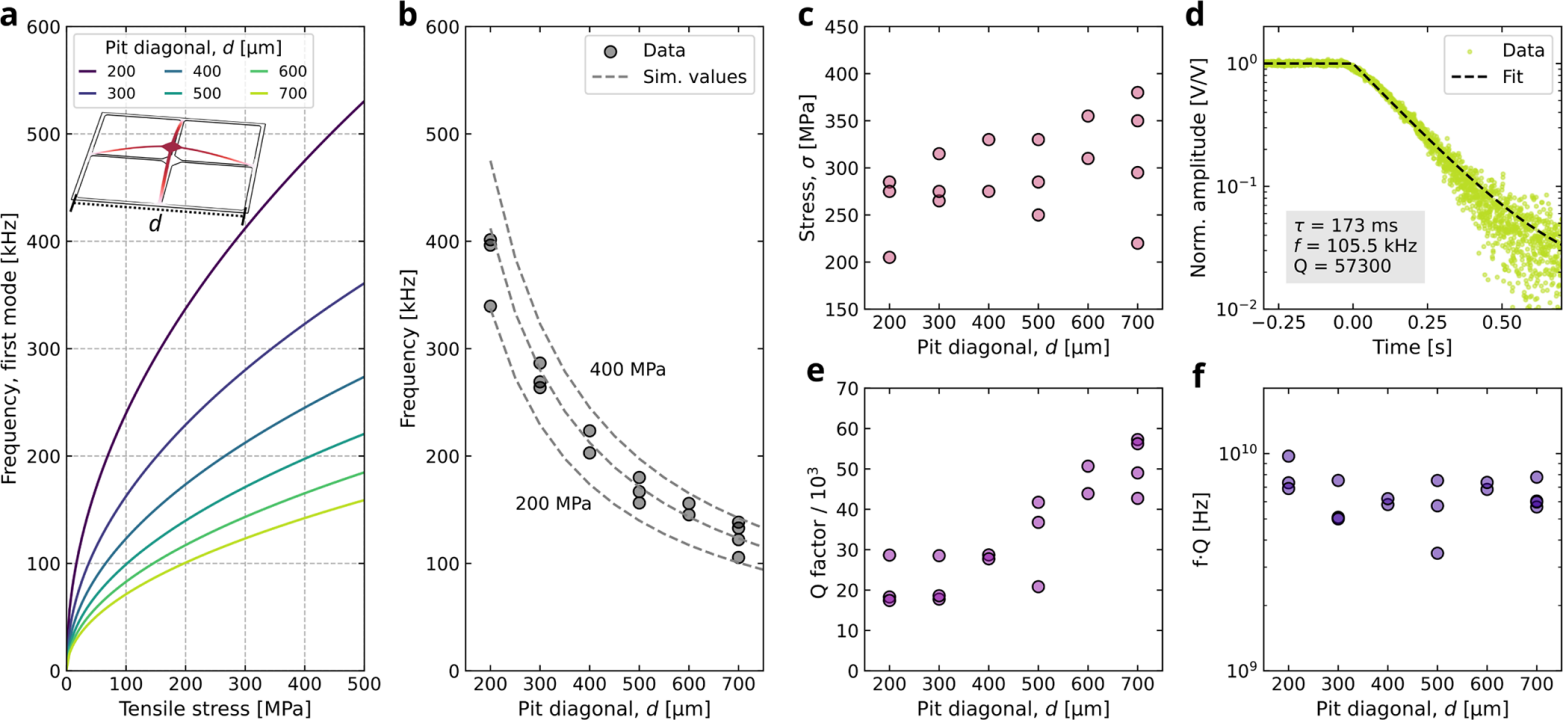
Mechanical characterization of LSMO trampolines. (a) Stress and size dependence of the first mode resonance frequency for LSMO trampolines calculated by finite element analysis. (b) Resonance frequencies of the first mode of LSMO trampolines. Dashed lines indicate the stress calculated from (a) and are spaced 100 MPa. (c) Isotropic in-plane tensile stress of the trampolines obtained by comparing the simulation results reported in (a) and the experimental data shown in (b). (d) Ring-down measurement of the first mode of the LSMO trampoline showing the highest measured *Q*-factor, having *d* = 700 μm. (e) *Q*-factor and corresponding f·Q products (f) as a function of the pit diagonal of the trampolines reported in (b).

### Magnetic Characterization

The magnetic properties of a pristine LSMO thin film grown on top of STO (110) were measured by a commercial DC-SQUID magnetometer (MPMS2 by Quantum Design, Inc. USA). Figure 3a shows the temperature dependence of the field-cooled magnetization *M*(*T*) with μ_0_ *H* = 50 mT applied along the [1 1® 0] direction, as indicated in the inset. This is the ″easy magnetization axis″ (see Supporting Information Section II for further details). The Curie temperature (*T*_C_) has been defined by the maximum of ∂ *M*/ ∂ *T* and it results in *T*_C_ = 344 ± 2 K, as expected for (La_0.7_,Sr_0.3_)­MnO_3_.^35^ The measured magnetization reaches 400 kA/m at 50 K. The onset of the magnetic transition can also be indirectly identified by measuring the temperature dependence of the electrical resistivity *R*(*T*), which shows a point of inflection just below the Curie temperature.^36^ In Supporting Information Section III, we compare the *R*(*T*) characteristics of the LSMO films employed for the different experiments discussed in this work, all showing similar *R*(*T*) behavior. The magnetization loop measured at room temperature (25 °C) is reported in Figure 3b,c. The saturation magnetization is above 250 kA/m, with about 170 kA/m of remanence and about 2.5 mT of coercivity.

**Figure 3:**
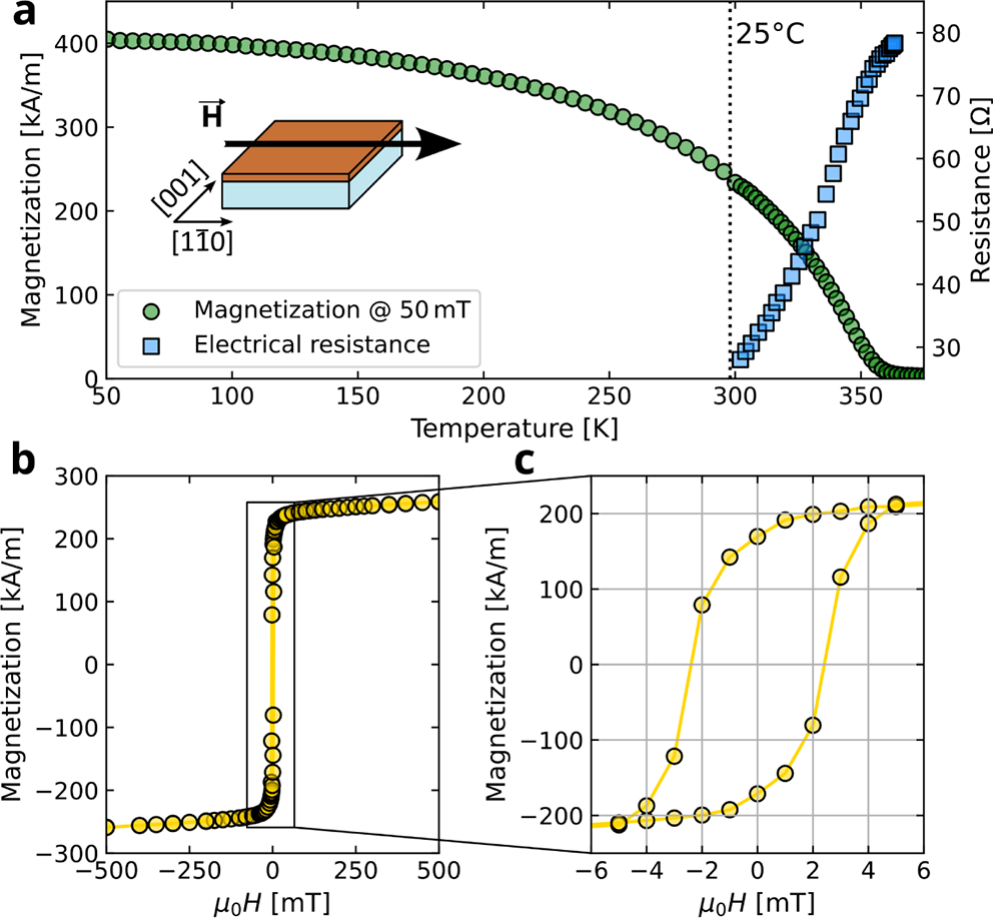
Magnetic characterization of LSMO(110) thin films. (a) Temperature dependence of the in-plane magnetization along the [11̅0] lattice direction (green dots) and normalized electrical resistance measured in four probe configurations (blue squares). (b) Magnetization loop at 25 °C measured in the ± 500 mT range. (c) Magnification of the data reported in (b) to better illustrate the magnetic hysteresis.

For our envisaged application, i.e., using TMO-based trampoline-shaped resonators as magnetic field sensors, we are also interested in their magnetic microstructure, which we investigated by magnetic force microscopy (MFM). Details of our specific MFM setup are described in the Experimental Section. For this experiment, we prepared a 100-nm-thick trampoline-shaped LSMO structure in a 240 μm × 280 μm^2^ frame with a 20 × 60 μm^2^ rectangular central pad and four 100-μm-long tethers. For the selected dimensions, shape anisotropy prefers an in-plane magnetization (typical for thin films) oriented along the long axis of the rectangle, i.e., the [11̅0] direction, which is also the easy axis of magnetization. This trampoline was not free-hanging, but was still attached to its growth substrate, i.e., MFM measurements were performed after dry etching the LSMO and the following cleaning of the surface. This was necessary because, in our setup, the interaction between the MFM tip and free-hanging trampolines made MFM data acquisition unreliable, often resulting in the destruction of the device. However, since the trampoline geometry retains its tensile stress, we do not expect a relevant difference in the micromagnetic configuration between the released and unreleased configurations.

The surface topography of the trampoline is reported in Figure 4a, showing almost all of the central pad and parts of the two bottom tethers, while the frame is not visible in this image. Figure 4b shows the pristine magnetic contrast in zero field. In the magnified inset, bright lines predominantly aligned along the long axis of the rectangle can be identified. In between these lines, the contrast is rather homogeneous. Note that a line-like contrast is also visible on the tethers. However, here we are mainly concerned with the domain structure on the central pad. Due to their geometry, MFM tips are mostly sensitive to the out-of-plane component of the stray field emanating from the sample. Thus, line-like features in MFM images of magnetic thin films indicate a domain-wall contrast and, thus, an in-plane magnetization of the sample. Moreover, a simple dark (or bright) line indicates the presence of Bloch-type domain walls, in which the magnetization in the domain wall rotates out-of-plane. We conclude that the observed MFM contrast is consistent with an in-plane magnetization with the easy axis of magnetization oriented along the [1 1® 0] direction. Figure 4c shows the remanent magnetic structure in zero field, i.e., after saturating the whole LSMO film in an in-plane external field of 20 mT applied along the long axis of the rectangle (actually, saturation is already observed at about 4 mT). In saturation, the contrast is homogeneous across the trampoline (it is in a single-domain state), with a dark and bright contrast at the opposite short sides of the central pad, where the magnetic field lines leave the sample pointing in and out of the sample plane. The first domains with an in-plane magnetization antiparallel to the direction of the saturating field start to nucleate as spike domains at the short edges of the rectangle. In remanence, they are only a few micrometer long and cover less than 3% of the area of the central pad. Upon reversing the direction of the external magnetic field, the domains grow further until the central pad is saturated in the opposite direction. This growth is displayed in the image series in Figure 4c. The easy axis of magnetization, saturation magnetization, domain nucleation, and growth visible in the MFM data are in good agreement with the measured *M*(*H*) hysteresis loop displayed in Figure 3b,c.

**Figure 4:**
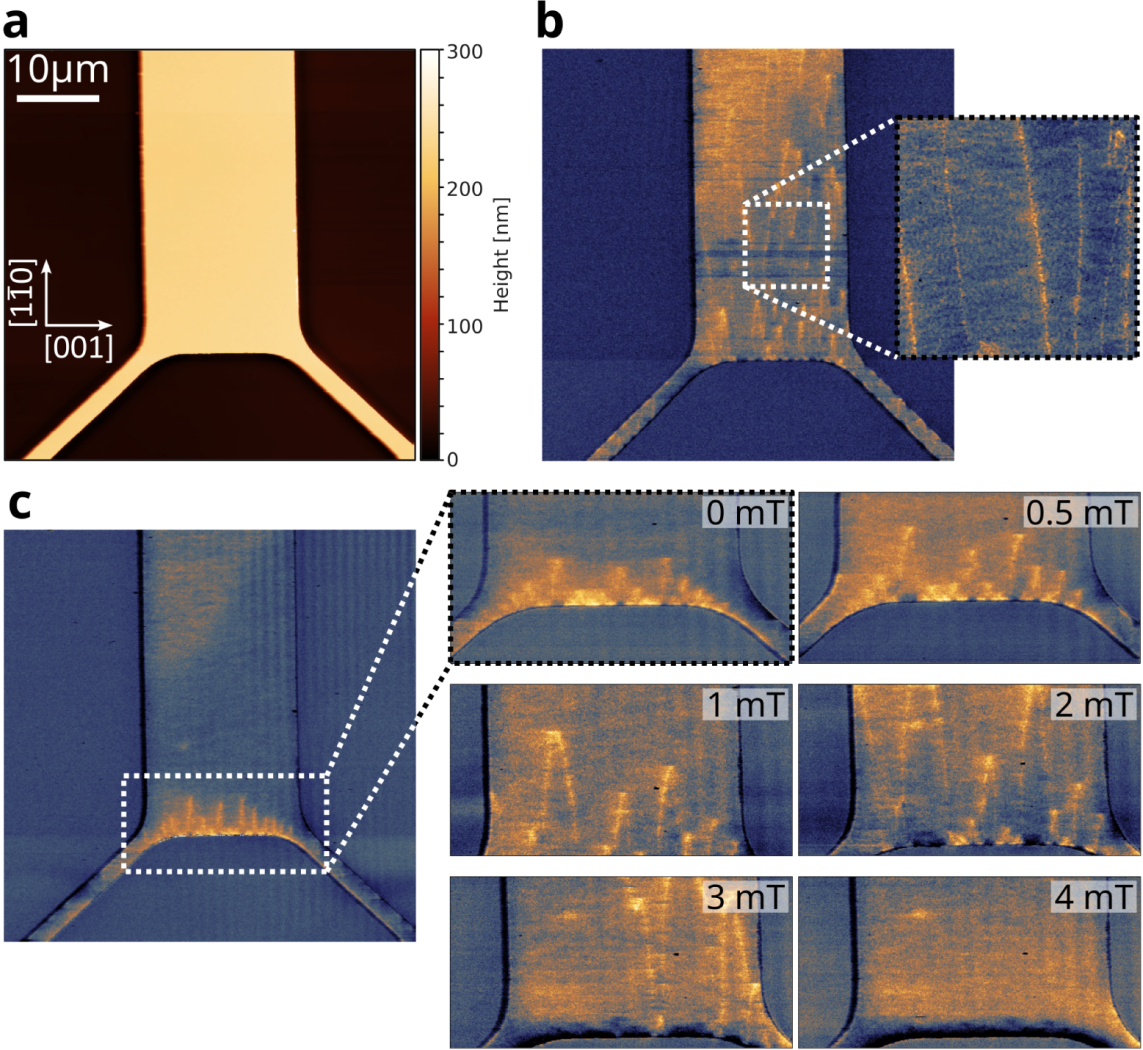
Micromagnetic characterization of LSMO(110) clamped trampolines. (a) Surface topography of a LSMO rectangular trampoline measured by atomic force microscopy using a magnetic tip. (b) Magnetic phase contrast of the region shown in (a) without premagnetization. Edges of magnetic domains are visible as lighter vertical stripes. (c) Magnetic microstructure after applying 20 mT in-plane. Magnified regions show the domains’ evolution while applying a magnetic field in the reverse direction.

## Conclusions

In conclusion, supended magnetic trampolines with diagonal lengths of up to 700 μm were realized from LSMO epitaxial thin films grown on SrTiO_3_(110) substrates. These resonators have in-plane tensile stress typically between 250 and 350 MPa and a mechanical quality factor of up to 60k, with signatures of dissipation-dilution-dominated mechanical losses. Room-temperature magnetic characterization of the bare film before microfabrication and of the patterned trampoline before suspension shows similar coercitivity of about 2.5 mT. The saturation magnetization at room temperature is 250kA/m, reaching 400kA/m at liquid nitrogen temperature. The key result of our work relies on its simplicity, as we obtain a micromechanical resonator with a relatively high-quality factor and net magnetic moment based on a one-step fabrication process. The combination of high mechanical quality factor and magnetic properties makes LSMO trampolines ideal candidates for realizing MEMS magnetic sensors, particularly when combined with other complex oxides in multifunctional heterostructured devices.

## Experimental Section

### (La,Sr)­MnO_3_ Thin Films Deposition

LSMO thin films are grown by pulsed laser deposition on top of non-terminated 5 × 5 mm^2^ single-crystal SrTiO_3_(110) substrates. The substrate is mounted in a stainless-steel sample-holder that is kept facing downward during the deposition. The growth chamber has a base pressure below 1 × 10^–8^ mbar, while during the growth, the oxygen pressure is 1 × 10^–4^ mbar. The KrF excimer laser (248 nm) energy density is 0.6 J cm^–2^, the laser repetition rate is 2 Hz, and the target–sample distance is about 45 mm. During the growth, the temperature is monitored by an infrared pyrometer measuring the backside of the sample holder. LSMO thin films implemented in this work were grown at 850 °C. The growth rate was 0.56 nm/min, as measured by monitoring the intensity oscillations of the reflection high-energy electron diffraction signal.

### Microfabrication

LSMO trampolines are realized by UV photolithography. The SPR-220-4.5 photoresist from Microposit is deposited by drop casting and spinning at a speed of 6000 rpm for 45 s. Resist baking is performed at 125 °C for 150 s. Exposed LSMO regions are etched in about 45 min by Ar^+^ ion milling with an energy of 500 eV and a current density of 0.2 mA/cm^2^. Then, the trampolines are suspended by immersion in 5% HF in water solution at 35 °C for about 1 h. During the corrosion step, the sample is kept above a magnetic stirrer spinning at about 200 rpm. At the end of the chemical etching step, the sample is immersed for about 30 min in a 5% H_3_PO_4_ aqueous solution to mechanically remove insoluble residues of the SrTiO_3_ corrosion in HF. This solution is kept at 40 °C and magnetically stirred at 100 rpm. When ready, the sample is cleaned by immersion first in water and then in ethanol baths. Finally, it is dried in an L-CO_2_ critical point dryer.

### Mechanical Measurements

Mechanical measurements were performed in a custom setup featuring active temperature control by a Peltier electro-thermal module. Samples were kept at were 25 °C, and the residual air pressure was below 1 × 10^–4^ mbar. Device motion was probed using the optical lever detection scheme with a four-quadrant photodiode. Mechanical excitation was provided by an AC-biased piezoelectric element glued by ceramic epoxy near the device.

### Finite Element Analysis

Numerical simulation of the trampoline’s resonances was performed in COMSOL Multiphysics v.6.2 using the membrane module. The model geometry consisted of a 20 μm^2^ squared pad with 5 μm tethers clamped to an external squared frame. Tether length and frame size varied according to the *d* parameter. The frame had a “Fixed” condition, while the rest of the edges were set as “Free boundary”. The membrane thickness was set to 100 nm. The mesh was generated using the “Physics-Controlled mesh” sequence type with the “Extremely fine” element size setting. Isotropic stress was imposed all over the membrane as an initial condition and allowed to relax in the suspended regions. The study type was “Prestressed eigenfrequency” with geometric nonlinearity enabled and a parametric sweep over the stress value and pit diameter (*d*). The simulation file is included in the data set (see “Open Data”).

### Magnetic Force Microscopy (MFM) Measurements

MFM was employed to probe the magnetic microstructure of an LSMO thin film with an unreleased trampoline geometry at room temperature. We used an MFM cantilever (resonance frequency: ∼75 kHz; spring constant: ∼3 N/m) with a hard magnetic coating and out-of-plane sensitivity. We used a Nanoscope IIIa instrument with a homemade calibrated dipole electromagnet (two copper coils in a Helmholtz configuration with permalloy yokes), which enables us to generate an in-plane magnetic field of about ±20 mT at the sample position. For imaging, we used the so-called lift mode with amplitude modulation (tapping mode). This mode of operation generates a topography image and an MFM image in one go. Both images are recorded line-by-line. First, the surface profile, i.e., the topography *z*(*x*,*y*), is recorded with active *z*-piezo feedback using the amplitude signal. Thereafter, the same line is recorded without feedback at a constant lift height of 80 nm above the surface by using the previously recorded topography data. The recorded variation of the phase signal ϕ­(*x*,*y*) is induced by the long-range magnetostatic tip–sample interaction, which reflects the magnetic microstructure of the sample. To minimize long-range electrostatic contributions to the phase signal, we compensated for the contact potential between the magnetic tip and the LSMO surface by applying a bias voltage of −1.8 V.

## Supplementary Material



## Notes

LSMO_trampolines-supplementary.pdf, containing: magnetization loops of LSMO(110); comparison of resistance vs temperature in LSMO(110) films; X-ray diffraction data of LSMO(110) films (PDF)
