# Supplementary material for: Magnetic Trampoline Resonators Made of (La,Sr)MnO3 Single-Crystal Thin Films
Source: ACS Sens. 2025 Jun 6;10(6):4244–50. doi: 10.1021/acssensors.5c00448 (PMC12339007; doi:10.1021/acssensors.5c00448)
Supplement: Supplementary file 1 [file se5c00448_si_001.pdf]

Supporting Information

Magnetic Trampoline Resonators made of (La,Sr)MnO_3 Single-Crystal Thin Films

Nicola Manca [1,*] Dhavalkumar Mungpara [2]

Leonélio Cichetto Jr [1] Alejandro E. Plaza [1] Gianrico Lamura [1]

Alexander Schwarz [2] Daniele Marré [3,1] and Luca Pellegrino [1]

[1]*CNR-SPIN, C.so F. M. Perrone, 24, 16152 Genova, Italy*

[2]*Institute of Nanostructure and Solid State Physics, University of Hamburg, 22761 Hamburg, Germany*

[3]*Dipartimento di Fisica, Università degli Studi di Genova, 16146 Genova, Italy*

This supplemental material contains the following:

- Section I: X-ray diffraction data of LSMO(110) films
- Section II: Magnetization loops of LSMO(110)
- Section III: Comparison of Resistance vs Temperature in LSMO(110) films

* nicola.manca@spin.cnr.it

Sec. I. X-RAY DIFFRACTION DATA OF LSMO(110) FILMS

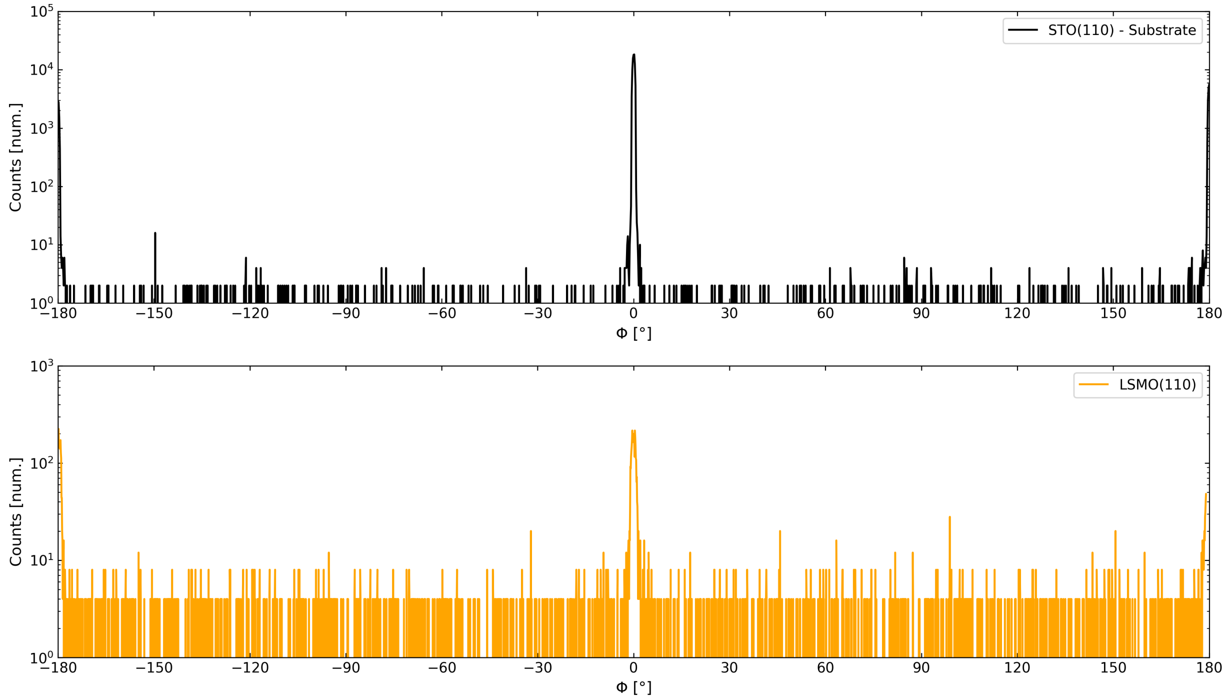

Figure S1. (top) ϕ-scans of the STO(110) substrate and (bottom) the LSMO(110) film [(013) peak]. The angular position of the diffraction peaks corresponding to the LSMO thin film and the STO substrate are identical. This indicates that the in-plane lattice structure of the LSMO film follows that of the underlying STO (epitaxial growth) which has 180° symmetry because of the sample surface corresponds to the (110) cut-plane.

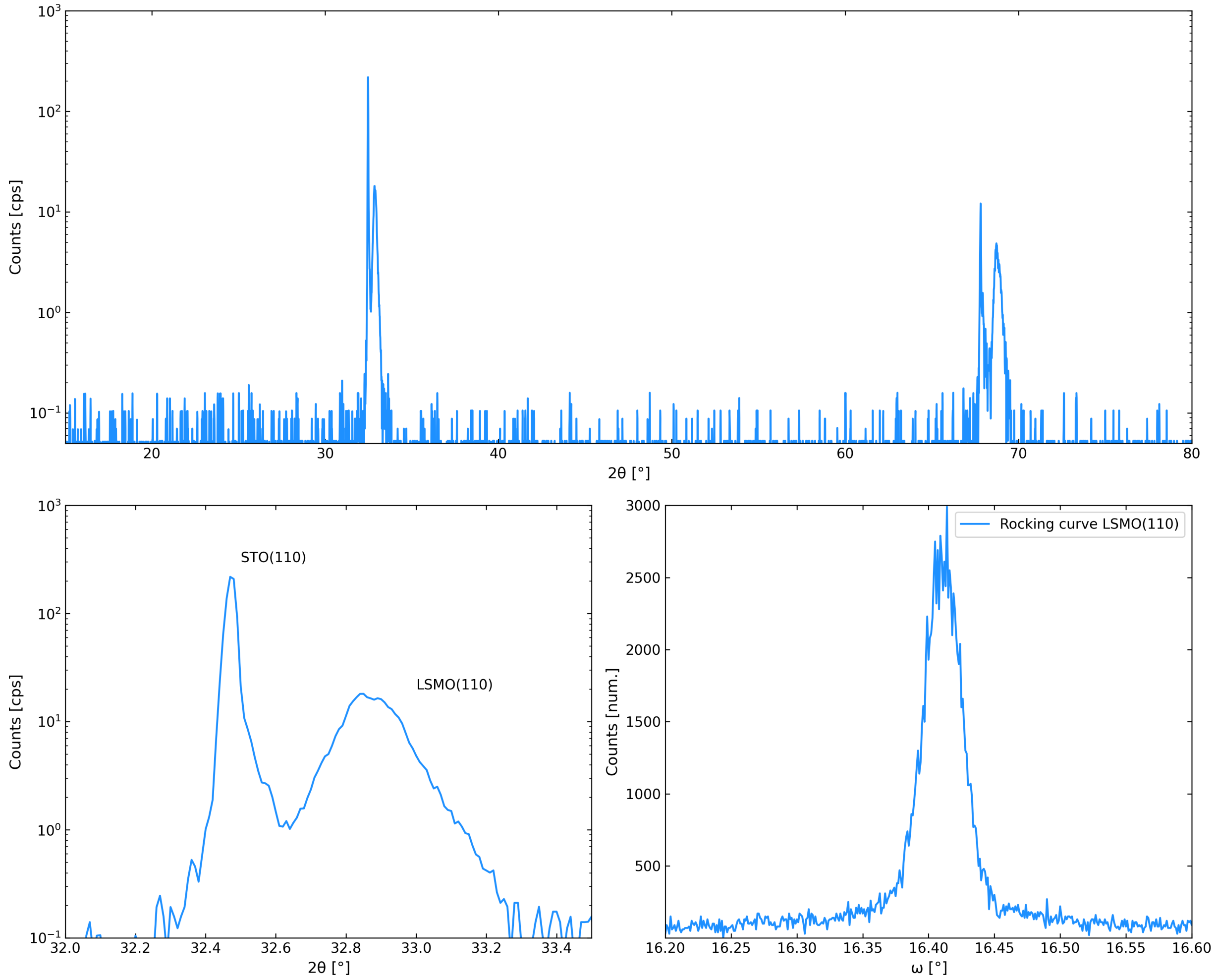

Figure S2. (top) θ–2θ scan of a LSMO thin film deposited on top of a STO(110) substrate and showing the (110) and (220) peaks of both of them. The absence of peaks other than those corresponding to the ($ll0$) reflections indicates absence of spurious phases. (bottom left) Magnification of the θ–2θ scan around the (110) peaks of STO and LSMO. (right) ω scan (rocking curve) of the LSMO film showing a full width at half maximum of about 0.04 degrees. This indicates low dispersion of the lattice planes in the out-of-plane direction, to be compared to the typical values measured for STO substrates of about .

Sec. II. MAGNETIZATION LOOPS OF LSMO(110)

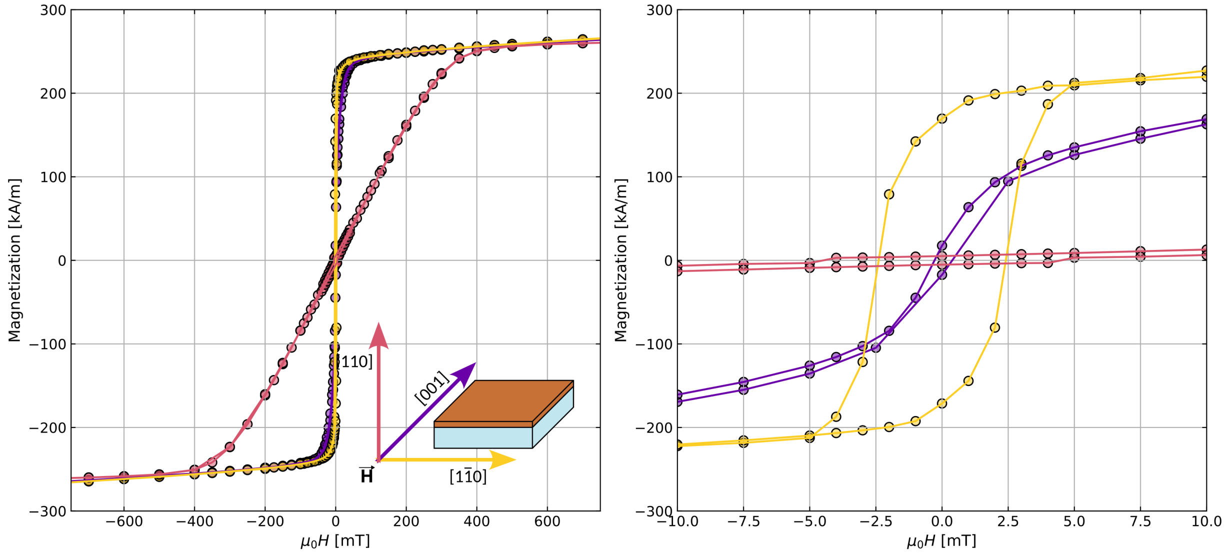

Figure S3. Room-temperature magnetization loops of a bare (La,Sr)MnO_3 film measured along the three main lattice directions, as show in the inset of the left panel. The right panel shows the same data but in a narrower range for better comparison. Above saturation the magnetization shows a small linear increase. The observed linear increase is independent of the field direction. We thus address this effect to a paramagnetic impurity present in the system that we could not identify.

Sec. III. COMPARISON OF RESISTANCE VS TEMPERATURE IN LSMO(110) FILMS

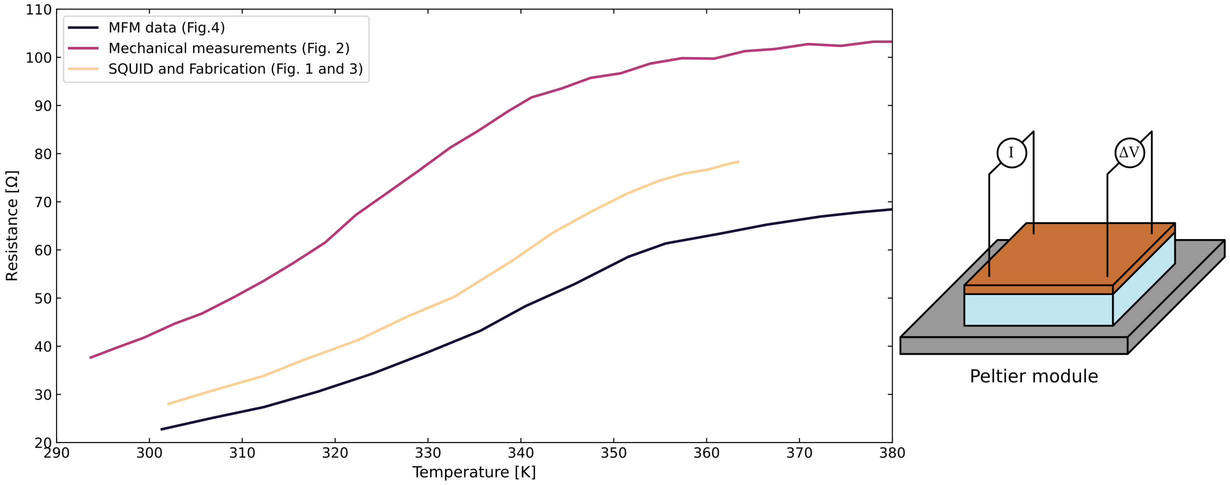

Figure S4. Resistance vs temperature characteristics of the LSMO thin films employed in this work. Plot labels indicate the use for each sample. The resistance measurements were performed in four-probes configuration with the sample in contact to a Peltier module to control its temperature. This simple measurement setup mimics Van der Pauw configuration but does not have the precision for contacts geometry required to obtain quantitative measurements. Its purpose is to provide a rough evaluation of the transport properties of deposited thin films and identify the transition temperature from the R(T) curve.
